# Deep Learning Optimisation Strategies for Uveal Melanoma Detection Using Ultra-Widefield Photography

**DOI:** 10.21203/rs.3.rs-9441188/v1

**Published:** 2026-05-11

**Authors:** Michael Heiferman, Sanjay Ganesh, Virginia Tasso, Reem AlAhmadi, Darvin Yi

**Affiliations:** University of Illinois at Chicago; University of Illinois at Chicago; University of Illinois at Chicago; University of Illinois at Chicago; University of Illinois at Chicago

## Abstract

**Objectives:**

Uveal melanoma (UM) is the most common primary intraocular malignancy in adults and carries significant metastatic risk. Early and accurate diagnosis is essential, but challenging due to overlapping clinical features with benign choroidal nevi. Deep learning (DL) offers potential to support early and accessible detection, but model performance is limited by dataset size and quality. This study evaluates data-centric optimisation strategies for DL classification of UM using ultra-widefield (UWF) fundus photography.

**Methods:**

This retrospective study analysed UWF fundus photographs from 784 patients (864 images) seen at the University of Illinois Chicago eye clinic. A baseline binary classification model (UM vs. choroidal nevus) was compared with seven models incorporating optimisation strategies across three categories: class addition, dataset augmentation, and enhanced feature selection. Performance was assessed using AUC, F1 score, precision, and recall, with calibration evaluated via expected calibration error.

**Results:**

The baseline model achieved an AUC of 0.906 ± 0.032. The top-performing model incorporated healthy retinal controls as an additional class, achieving an AUC of 0.987 ± 0.011 and F1 scores of 0.966 (nevus) and 0.941 (UM). Dataset augmentation approaches yielded minimal performance gain, and Multi-strategy models showed no additive benefit.

**Conclusions:**

Data-centric optimisation significantly influences DL performance for UM detection. Three principles emerge: healthy class addition improves specificity and anatomical feature learning; data quality may outweigh quantity; and contextual input tuning is a key model parameter. These findings offer a practical framework for developing clinically robust, physician-supervised AI tools to support early UM triage and reduce diagnostic variability.

## Introduction

Uveal melanoma (UM) is the most common primary intraocular malignancy in adults.^1^ Each additional millimetre of tumour growth is associated with a significant increase in metastatic risk, ranging from 6% to 51% depending on tumour thickness.^2^ Therefore, early detection is essential for improving survival; however, this can be challenging as UM often mimics benign lesions such as choroidal nevi or congenital hypertrophy of the retinal pigment epithelium (CHRPE). Accurate diagnosis at presentation is critical, as these conditions carry very different management plans. UM is typically treated with radiation or enucleation, whereas benign lesions are monitored for growth and potential malignant transformation.^3,4^

Traditional diagnostic workflows for choroidal lesions rely on multimodal imaging and clinical evaluation.^5–7^ Modalities such as ultra-widefield (UWF) fundus photography, ultrasonography, and optical coherence tomography are used to assess tumour characteristics and distinguish benign from malignant lesions. One drawback of current practice is that image interpretation varies widely among clinicians and relies heavily on individual expertise to discern complex and subtle imaging features. These challenges are amplified in regions with limited access to specialised ocular oncologists, contributing to delayed diagnosis, continued tumour growth, and poorer prognosis.^8^ Given the aggressive nature of UM and its high metastatic potential, the absence of rapid, objective, and accessible triaging tools remains a critical gap in current care. Automated, clinician-supervised diagnostic tools could help address this need by supporting early detection and risk stratification of UM, reducing delayed intervention and over-referral.

Artificial intelligence (AI)-assisted tools have shown potential in the detection and management of UM. Deep learning (DL), a subset of AI, enables models to learn patterns from data without explicit programming and is widely used in clinical AI research. Prior work in this space has investigated optimal model architectures, parameters, and downstream task fine-tuning.^9–16^ While these studies showcase promising results, model performance is also strongly influenced by the composition and quality of training data.^17^ This is particularly relevant in low-data clinical settings, such as UM detection, where limited and imbalanced datasets can constrain model convergence and generalisability. The impact of data-centric optimisation strategies remains underexplored, and a clearer understanding of how these strategies influence model performance may help guide the development of robust tools ready for clinical use.

This study shifts focus from architectural choices to input data composition, examining how dataset construction and input design shape DL model behaviour in UWF-based UM classification. We evaluate the effects of class addition, dataset augmentation, and enhanced feature selection on binary classification of UM versus nevus, assessing their influence on discriminative ability and calibration. By identifying the most effective data-centric strategies, this work establishes practical design principles for early UM detection and offers a transferable framework for clinical DL development in other data-limited settings.

## Methods

### Dataset

This retrospective study analysed UWF fundus photographs (Optos PLC, Dunfermline, Fife, Scotland, UK) to develop DL models for early UM detection. UWF imaging was selected for its broad field of view, improved visualization of peripheral lesions compared with traditional color fundus photography, and widespread availability across diverse clinical settings. This study adhered to the Declaration of Helsinki and obtained IRB approval from the University of Illinois Chicago. Informed consent was waived due to the retrospective design. A total of 784 unique patients were included, contributing 864 UWF images across four classes: UM (170), choroidal nevus (290), CHRPE (102), and healthy controls (302) (Table 1). All patients were seen at the University of Illinois Chicago Eye and Ear Infirmary between January 2010 and October 2025.

The inclusion criteria were patients older than 17 years with available UWF fundus imaging from their initial presentation to our clinic. Poor quality images, such as those that did not visualise a majority of the tumour, those with previously treated tumours, or those containing multiple lesions, were excluded. Images were labelled based on clinical diagnosis by an ocular oncologist using ophthalmoscopy, ultrasonography, fundus photography, fundus autofluorescence, optical coherence tomography, and fluorescein angiography at the clinician’s discretion. When available, histopathologic confirmation from enucleation specimens or biopsy was used to confirm diagnoses. Indeterminate lesions were monitored longitudinally; those that demonstrated growth or required treatment were classified as melanoma. No minimum follow-up period was required to exclude slow-growing melanomas, as the study aimed to evaluate performance based on diagnostic assessment at the time of presentation. All data were accessed between September 2022 and November 2025.

### Study Design and Model Development

**Figure 1:**
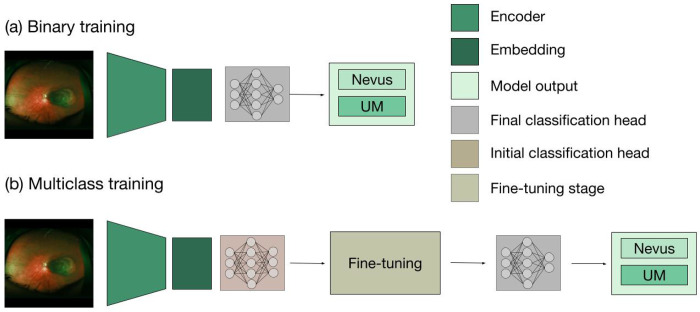
High-level overview of the deep learning architecture A) Standard architecture for binary training. B) Additional fine-tuning step illustrated for multi-class models to enable validation on a two-class dataset.

Eight DL models were developed using the ResNet-50 architecture as the feature extractor backbone to ensure fair comparison across experiments. The ResNet-50 architecture was chosen because it is a well-validated benchmark in computer vision, providing a strong foundation for all experiments.^18, 19^ An ImageNet-pretrained backbone was employed to leverage already learned general image features such as edges and contours. A task-specific classifier was subsequently appended to match the number of output classes in each experiment. Images were resized to 224 x 224 pixels to match the ResNet-50 pretraining input resolution. A baseline binary classification model (UM versus choroidal nevus) was first developed using UWF fundus photographs at initial presentation. A high-level schematic of the model architecture used in this study is illustrated in Fig. 1a. Subsequent experiments systematically introduced different data configurations, and the performance of each model was compared against the baseline and one another. Optimisation strategies were divided into three categories: class addition, dataset augmentation, and enhanced feature selection. Two final multi-strategy models were developed to evaluate interactions between different optimisation strategies.

Two models were developed in the class addition group. The first model introduced CHRPE as a third class to evaluate whether expanding class diversity with a well-demarcated lesion improved model discrimination. The second model introduced a healthy control class, which included UWF photographs without any fundus lesions. The inclusion of this class was intended to expose the model to examples of typical retinal anatomy, helping it learn more robust representations of background texture, structure, and imaging artifacts.

Two dataset augmentation strategies were explored to increase the diversity of training data and improve robustness. The first was the inclusion of multiple UWF fundus photographs per patient, when available, provided images were acquired on different dates or offered unique tumour perspectives. This ‘multi-optos’ experiment included an additional 194 and 536 in the UM and nevus classes, respectively, bringing the training totals to 364 UM and 826 nevus images. The second experiment included post-treatment UM images, which added 31 images to the UM class, bringing the total to 201 UM images. In both experiments, additional images were used exclusively during training and were excluded from validation and test sets, and additional steps were taken to ensure proper patient-level stratification to prevent data leakage.

A single model was developed in the enhanced feature selection category: a region of interest (ROI) dilation model. A fundamental challenge in image classification is the need to downsize images, which does not preserve minute details useful for accurate differentiation in borderline cases. To test whether lesion-centered cropping before resizing improves performance, lesion boundaries were manually segmented by an ocular oncologist and used to generate ROI crops (Supplemental Fig. 1). This procedure serves two purposes: 1) to reduce the proportion of pixels used on non-informative background data, which may mask the useful discriminatory signal in the pixels belonging to the object of interest (the lesion), and 2) to preserve image detail by cropping before resizing, such that fewer relevant pixels are lost during downsampling yielding an improved resolution of the tumour. To evaluate the effect of contextual information surrounding the tumour, we applied dilation operations at multiple scales to generate a series of dataset variants in which images included increasing perilesional tissue data. An independent ResNet-50 model was trained on each dataset variant, enabling a controlled evaluation of how different amounts of contextual input influence performance and allowing identification of an optimal lesion mask dilation for this task. The highest-performing dilation configuration was subsequently compared against the other optimisation strategies evaluated in this study.

Lastly, two multi-strategy models were developed. The first integrated all strategies across the three optimisation categories, while the second incorporated only the top-performing (i.e., ‘best’) strategy from each category.

All experiments were conducted in Python (v3.8) using the PyTorch (v1.12.1) framework. Model training used an NVIDIA RTX 5000 series GPU with 32GB of VRAM. We used a 70/15/15 split for the dataset for the training, validation, and test sets, respectively. The split was done on a per-patient basis to avoid data leakage across sets. For each experiment, the training and validation data varied according to the optimisation strategy, while the test set was held constant between all models to ensure fair comparison. Specifically, all models were compared on the same binary UM and nevus discrimination task using the same validation set, regardless of optimisation strategy. The only exception was models incorporating the ROI-dilation strategy, for which lesion-centred cropping was applied to input images.

Independent hyperparameter tuning was conducted using the Optuna framework for each model to ensure performance differences reflected optimisation strategy rather than suboptimal tuning.^20^ For each model, the following hyperparameters were tuned: batch size, learning rate, dropout rate, and weight decay (Supplemental Table 1). All models were trained using the Adaptive Moment Estimation (Adam) optimiser. The best model was selected based on the lowest validation loss.

As two models were initially trained on multiclass datasets, an additional fine-tuning phase was performed to adapt the classifiers to the binary task required at the final inference stage. This consisted of modifying only the final classifier of the model and performing a short fine-tuning phase with a reduced learning rate on a dataset comprising UM and nevus only, allowing the models to specialise in the desired binary classification task while preserving the encoder’s learned multiclass feature representations. This additional step is illustrated in Fig. 1b.

### Evaluation Metrics

Model performance was primarily evaluated using the F1 score and Area Under the Curve (AUC). Two F1 scores were reported (with each class as the positive class) to account for the asymmetry introduced by class imbalance and to ensure that aggregate metrics did not obscure performance on the minority class. The AUC quantifies a model’s discriminative ability across all possible decision thresholds, providing a threshold-independent measure of ranking performance. Precision and recall were also reported by class to enable a more granular analysis of each model.

In addition to discrimination metrics, the expected calibration error (ECE) was computed to evaluate the calibration of probabilistic predictions in the presence of class imbalance. The ECE measures the discrepancy between predicted probabilities and observed event frequencies by grouping predictions into bins and comparing average predicted risk to empirical outcomes within each bin. Lower values indicate better calibration, meaning predicted probabilities more closely align with true outcome frequencies and can be more reliably used for clinical risk stratification. Bootstrapping was performed with 1 000 resampling iterations at the patient level to derive empirical 95% confidence intervals.

## Results

A total of 784 unique patients diagnosed with UM, choroidal nevi, CHRPE, or healthy controls were included in this study. The mean age and sex ratios were comparable between groups. The demographic information and clinical characteristics of the lesions are summarised in Table 1.

**Figure 2:**
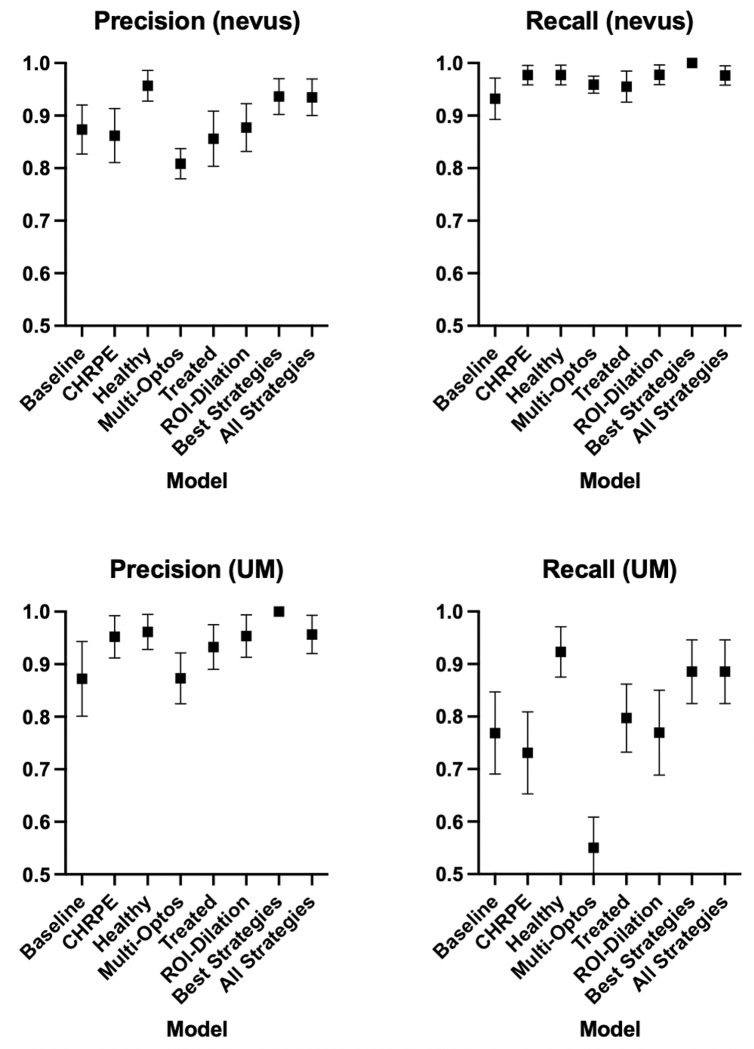
Bar charts of precision and recall scores by class and by model

The classification performance for all models is summarised in Table 2. The baseline model achieved an AUC score of 0.906 ± 0.032, F1 (nevus) of 0.901 ± 0.033, and F1 (UM) of 0.816 ± 0.059. The best-performing models were the healthy class addition model (AUC: 0.987 ± 0.011; F1 (nevus): 0.966 ± 0.020; F1 (UM): 0.941 ± 0.035), the ‘all-strategies’ model (AUC: 0.963 ± 0.027; F1 (nevus): 0.978 ± 0.015; F1 (UM): 0.960 ± 0.026), and the ‘best-strategies’ model (AUC: 0.950 ± 0.033; F1 (nevus): 0.945 ± 0.023; F1 (UM): 0.898 ± 0.046). The multi-optos model was the only model to display decreased performance compared to the baseline model, with an AUC of 0.814 ± 0.035, F1 (nevus) of 0.877 ± 0.019, and F1 (UM) of 0.678 ± 0.049. Precision and recall for the UM and nevus groups are shown in Fig. 2. Notably, the best-strategies model achieved a precision of 1.0 for UM and a recall of 1.0 for nevus, indicating zero false positives for UM and zero missed nevi.

To evaluate overall accuracy and calibration of probabilistic predictions, we computed the ECE. The baseline model achieved an ECE of 0.130 ± 0.028. All models achieved superior calibration to the baseline model, with ECE scores below 0.130. The best-calibrated models were the healthy class addition and both multi-strategy models, with the healthy model achieving the best overall calibration, with an ECE of 0.049 ± 0.018. All ECE scores are illustrated in Table 2.

## Discussion

DL-based approaches for early detection of UM have shown considerable promise in recent years. While various architectural designs and optimisations have been studied, data-centric optimisations remain poorly characterised despite potential for improvement. In this study, we systematically evaluated how different data-centric optimisation strategies affect DL model performance for UM detection using UWF fundus photography. Several strategies produced meaningful improvements in discriminative performance and calibration, while others revealed important failure modes. Together, these findings offer a principled framework to guide future model development in this and related rare-disease classification settings.

Several strategies proved particularly effective in improving feature abstraction and discrimination between UM and nevi. The three highest-performing models were the healthy class addition, best-strategies (healthy addition, treated UM inclusion, and ROI dilation), and all-strategies models, achieving AUC scores of 0.99, 0.95, and 0.96, respectively. The substantial performance and calibration gains observed with the inclusion of healthy retinal controls point to an important principle: healthy-class grounding. By exposing the model to examples of normal retinal anatomy, the training process appears to anchor the model’s feature representations in what the background should look like in the absence of pathology. Without this grounding, the baseline model may have partly relied on background texture and imaging artifacts as proxy signals for class discrimination. This is supported by Grad-CAM activation maps (Fig. 3). The baseline model displays diffuse, nonspecific activations across healthy tissue, whereas the best-strategies model concentrates activation at diagnostically relevant regions, such as lesion margins and high-contrast borders. Grounding through healthy class addition, therefore, functions as an implicit normaliser, redirecting model attention away from spurious background correlations and towards true lesion-specific features.^21^

The ROI dilation experiments establish a second principle: model performance is sensitive to lesion context, and an optimal ROI dilation balances the underlying biology of the diagnostic task without introducing too much irrelevant data. Progressive dilation of the lesion mask revealed an optimal tradeoff between perilesional context and classification performance, with neither the tightest crop nor the most expansive field of view yielding the best results. The best-performing configuration corresponded to a dilation in which the additional perilesional margin was equivalent to 50% of the original lesion mask area, capturing diagnostically relevant features such as surrounding pigmentation, subretinal fluid, and drusen. Notably, the performance gain from dilation was substantially greater for F1 (UM) than for F1 (nevus). This asymmetry is likely since UM lesions are on average larger than nevi, meaning a fixed proportional dilation yields a greater absolute expansion of the input and exposes the model to more perilesional context. For small nevi, the same proportional dilation adds comparatively less contextual tissue. This pattern suggests that DL-based UM classification has a contextual window aligned with diagnostic criteria used by clinicians, which can be balanced to optimise performance, computational resources, and human annotation effort. Contextual input should therefore be treated as a tunable hyperparameter informed by clinical knowledge rather than defaulting to full-image or arbitrary fixed-crop inputs.

Not all optimisation strategies improved overall performance. The multi-optos experiment was the only strategy to decrease performance relative to baseline (AUC 0.814 versus 0.906), illustrating that data quantity does not reliably translate to performance gains in medical imaging AI, an idea consistent with existing literature on language models.^17^ The addition of longitudinal images from existing patients increased the total training set size by over 250%, but introduced two compounding problems. First, multiple images from the same patient, even across timepoints, share lesion morphology, retinal anatomy, and imaging artifact profiles, increasing intra-class redundancy rather than true feature diversity. Additionally, not all additional images met the original quality threshold, introducing noise that may have destabilised learned representations. In contrast, the model trained with treated images showed a modest increase in performance, particularly in F1 (UM). This is likely due to the additional images in the UM group. Despite the morphological changes to the tumour from treatment, the additional data may have helped with discrimination, and the mitigated class imbalance helps explain the superior calibration.

The addition of CHRPE as a third class produced similarly limited gains, despite introducing genuine structural diversity. CHRPE lesions are characterised by sharply demarcated, densely pigmented borders that can differ significantly from nevi and UM. This suggests that lesion diversity alone is insufficient to drive meaningful improvement when the added class is less clinically or visually confusable with the target classes. Taken together with the multi-optos findings, these results indicate that the most valuable class additions are clinically adjacent examples that help the model resolve overlapping or ambiguous features. When expanding training datasets, researchers should therefore prioritise task-relevant diversity (e.g., variations in lesion morphologies, imaging conditions, and patient demographics) while maintaining appropriate class balance, rather than simply increasing dataset volume.

Beyond discrimination, calibration is a clinically critical property of diagnostic AI tools. In UM triaging, poor calibration carries direct clinical risk, as an overconfident model may assign high certainty to ambiguous lesions, potentially suppressing appropriate clinical concern or referral. Across our experiments, calibration performance did not uniformly track discrimination, with strategies that improved AUC producing only modest ECE gains, and others yielding improvements in both metrics. The ROI-dilation model, despite yielding meaningful gains in discriminative performance, did not produce a corresponding improvement in calibration over baseline. This is consistent with the nature of the optimisation strategy, as perilesional context introduces additional features enhancing discrimination but does not address the underlying class imbalance. In contrast, healthy class addition improved both discrimination and calibration, likely because exposure to normal retinal anatomy provides an explicit reference distribution that better captures non-pathological variability. This reduces the tendency to assign high-confidence predictions to ambiguous or background regions, resulting in probability estimates that more accurately reflect true uncertainty. This distinction highlights that discrimination and calibration respond to different aspects of data-centric optimisation and should be evaluated and targeted independently based on the anticipated use case.

The two multi-strategy models together offer insight into how individual optimisation gains interact when combined. The best-strategies model demonstrated non-additive performance, with performance slightly under the healthy class addition model. The all-strategies model curiously showed marginal improvements over the best-strategies model, despite incorporating strategies that may have increased noise. Notably, the healthy model (AUC 0.99), which applied a single strategy, outperformed both multi-strategy combinations, suggesting that the contribution of contextual grounding is the dominant optimisation. This finding argues against assuming that strategy stacking is always beneficial and supports a more selective, hypothesis-driven approach to data-centric optimisation.

This study has several limitations. This is a single-centre retrospective analysis, and external validation on independent datasets from other institutions with different device manufacturers and acquisition protocols is necessary to robustly assess generalisability. Importantly, the overall dataset size remains modest relative to large-scale DL benchmarks, and the relatively wide confidence intervals observed across experiments reflect this constraint. Confidence intervals were derived using bootstrap resampling, which is known to overestimate variance when used with small sample sizes, underscoring the need for larger, externally validated cohorts. Additionally, while class imbalance was assessed through calibration metrics, its influence on model behaviour cannot be fully disentangled from other factors. An important limitation is that the ground truth labels used were derived from clinical diagnoses at the time of initial presentation, which are subject to inter-physician variability; more objective definitions, such as genetic profiles or disease-specific mortality, may strengthen label reliability, though these are not routinely available. Future work should incorporate multicentre datasets, prospective validation, and exploration of optimisations for other clinically relevant models, such as semantic segmentation. Integrating multimodal imaging data and additional explainability techniques could further enhance clinical interpretability and trust.

This study moves beyond performance benchmarking to offer data-centric strategies to guide future model development in ophthalmic classification problems. Three principles emerge from our analyses. First, normative grounding through the inclusion of healthy retinal controls substantially improves feature specificity by redirecting model attention from spurious background correlations towards true lesion features. Second, contextual input is a meaningful and tunable hyperparameter with an optimal lesion-to-context ratio that should be informed by clinical knowledge of perilesional diagnostic features. Third, data quality and feature diversity matter more than sheer volume in certain cases, as indiscriminate dataset expansion through redundant or lower-quality images can degrade performance. These principles may not be specific to binary UM classification and may offer a transferable framework for data-centric DL development in rare-disease image classification more broadly. With multicentre validation and integration into clinical workflows, models informed by these design principles have the potential to reduce variability in interpretation, support earlier intervention, and ultimately save patient lives.

## Supplementary Material

This is a list of supplementary files associated with this preprint. Click to download.

- SupplementalTable1.png
- SupplementalFigure1.png
- Table1.jpg
- Table2.png
